# Construction of Minigenome Replicon of Nipah Virus and Investigation of Biological Activity

**DOI:** 10.3390/v17050707

**Published:** 2025-05-15

**Authors:** Fan Wang, Ruyi Chen, Jiayi Zhong, Anqi Zhou, Ran Peng, Bao Xue, Yuan Zhou, Jielin Tang, Xinwen Chen, Qi Yang

**Affiliations:** 1 Guangzhou National Laboratory, Guangzhou 510530, China; wang_fan2@gzlab.ac.cn (F.W.); chen_ruyi@gzlab.ac.cn (R.C.); zhong_jiayi@gzlab.ac.cn (J.Z.); xue_bao@gzlab.ac.cn (B.X.);; 2 GMU-GIBH Joint School of Life Science, Guangzhou Medical University, Guangzhou 511436, China; 3 Wuhan Institute of Virology, Chinese Academy of Sciences, Wuhan 430071, China; 4 State Key Laboratory of Respiratory Disease, Guangzhou Medical University, Guangzhou 510182, China; 5 The First Affiliated Hospital, Guangzhou Medical University, Guangzhou 510120, China

**Keywords:** Nipah virus (NiV), minigenome replicon, inclusion bodies (IBs), antivirals

## Abstract

Nipah virus (NiV), a highly lethal zoonotic pathogen causing encephalitis and respiratory diseases with mortality rates up to 40–70%, faces research limitations due to its strict biosafety level 4 (BSL-4) containment requirements, hindering antiviral development. To address this, we generated two NiV minigenome replicons (Fluc- and EGFP-based) expressed via helper plasmids encoding viral N, P, and L proteins, enabling replication studies under BSL-2 conditions. The minigenome replicon recapitulated the cytoplasmic inclusion body (IB) formation observed in live NiV infections. We further demonstrated that IB assembly is driven by liquid–liquid phase separation (LLPS), with biochemical analyses identifying the C-terminal N core domain of the N protein, as well as N_0_ and XD domains and the intrinsically disordered region (IDR) of the P protein, as essential structural determinants for LLPS-mediated IB biogenesis. The targeted siRNA silencing of the 5′ and 3′ untranslated regions (UTRs) significantly reduced replicon-derived mRNA levels, validating the regulatory roles of these regions. Importantly, the minigenome replicon demonstrated sensitivity to type I/II/III interferons and antivirals (remdesivir, azvudine, molnupiravir), establishing its utility for drug screening. This study provides a safe and efficient platform for investigating NiV replication mechanisms and accelerating therapeutic development, circumventing the constraints of BSL-4 facilities while preserving key virological features.

## 1. Introduction

Nipah virus (NiV) is responsible for highly lethal diseases, with case–fatality rates spanning from 40% to 70% in identified outbreaks [1]. NiV, an RNA virus, is a member of the *Paramyxoviridae* family and the *Henipavirus* genus [2] and classified as a Biosafety Level 4 (BSL-4) agent. There are currently no treatments or licensed vaccines available for the prevention and control of NiV infections [3]. The NiV genome consists of six transcription units arranged as 3′-N-P-M-F-G-L-5′. These units encode three non-structural proteins (C, V, and W) and six viral structural proteins: the nucleoprotein (N), phosphoprotein (P), matrix protein (M), fusion glycoprotein (F), attachment glycoprotein (G), and large polymerase (L). The N encapsidates the genomic RNA of NiV, forming a long helical nucleocapsid structure, which serves as a template for the replication and transcription of the viral genome, and the P and L proteins form the viral polymerase complex, which are essential for NiV replication [4,5,6,7].

Research on highly pathogenic viruses such as NiV [8], Ebola virus (EBOV) [9], and Marburg virus (MARV) [10] is limited because live virus experiments require BSL-4 laboratories. The minigenome replicon has provided researchers with the opportunity to study highly pathogenic viruses under BSL-2 conditions [11,12]. In the *Paramyxoviridae* family, the minigenome contains promoters for T7 polymerase, RNA polymerase I, or RNA polymerase II, flanked by a viral 5′ untranslated region (5′ UTR) and 3′ untranslated region (3′ UTR), a reporter gene, and corresponding terminators. Upon transfection into host cells, the minigenome is transcribed by the corresponding RNA polymerases to generate the negative-sense reporter gene RNA. Subsequently, the viral replication machinery, including P and L, utilizes the encapsidated negative-sense RNA as a template to synthesize positive-sense RNAs. These newly synthesized RNAs are then translated by host ribosomes to produce reporter proteins (e.g., luciferase or EGFP), allowing the quantitative assessment of viral replication efficiency [13]. In studies on NiV minigenome replicon, Kim et al. developed a four-plasmid-based NiV minigenome replicon capable of expressing the chloramphenicol acetyltransferase (CAT) reporter gene in normal African green monkey kidney fibroblast cells (CV-1) [14]. However, the system failed to validate its functionality in human lung cells and nerve cells, which are the target tissues for NiV, thereby limiting its applicability in studies of viral transcription and replication regulation. Recently, Ke et al. developed a stable NiV minigenome replicon using helper cells expressing NiV N, P, and L proteins, but it was not insensitive to small-molecule inhibitors, limiting utility for drug screening [11]. Therefore, the development of novel minigenome replicon systems remains critically relevant.

The replication and assembly of many viruses occur in specialized intracellular compartments known as viral factories, viral inclusions, or viroplasms [15,16]. In the case of positive-strand RNA viruses, viral factories are associated with rearrangements of membranes from diverse organelles (mitochondria, endoplasmic reticulum, and other cellular organelles) leading to the formation of double-membrane vesicles (DMVs) [17]. In contrast, negative-strand RNA viruses induce the formation of membrane-less cytoplasmic inclusion bodies (IBs), such as Rabies virus [17], MARV [18], and respiratory syncytial virus (RSV) [19]. The IBs are responsible for the transcription and replication of the viral genome and consist mainly of the viral ribonucleoproteins (vRNPs). Additionally, many negative-strand RNA virus-induced membrane-less compartments and organelles have been proposed to assemble through liquid–liquid phase separation (LLPS), e.g., RSV [20], Measles virus (MeV) [21], and EBOV [22]. Previous studies have documented that NiV IBs exhibit liquid properties, demonstrating dynamic fusion capabilities in infected cells [23]. However, the formation of IBs driven by LLPS requires further investigation.

In this study, we generated an NiV minigenome replicon in which the proteins N and P in tandem are co-expressed via Porcine teschovirus-1 2A peptide (P2A). The NiV Fluc minigenome replicon is able to mimic the IB-like structures formed by virus-infected cells in the NiV-sensitive cells, demonstrating for the first time that NiV IBs are formed by LLPS-driven formation. Important, the new minigenome replicon is sensitive to interferons and antivirals, enabling drug screening.

## 2. Materials and Methods

### 2.1. Plasmid Constructions

The NiV minigenome was constructed according to the method previously described [24]. The minigenome replicon backbone, extending from the 5′ end to the 3′ end, comprises a T7 promoter, a 3′ non-coding tail region sequence, the 5′ UTR of the N gene, a reporter gene, the 3′ UTR of the L gene, a 5′ leader sequence, a hepatitis delta virus ribozyme, and a T7 terminator. The backbone fragment was assembled with the firefly luciferase (Fluc) gene or enhanced green fluorescent protein (EGFP) to generate the minigenome plasmid. The pCAGGS vectors carrying NiV N, P, and L genes were kindly provided by Professor Xiaoli Xiong from Guangzhou Institutes of Biomedicine and Health, Chinese Academy of Sciences. The pCAGGS-NiV-N/P was constructed by fusing the N and P proteins via a P2A self-cleaving peptide.

The plasmid pCAGGS-NiV-N-mCherry was obtained by subcloning the sequence encoding mCherry in fusion with the 3′ end of the N gene. The plasmid pCAGGS-NiV-P-EGFP was obtained by subcloning the sequence encoding EGFP in fusion with the 3′ end of the P gene. A series of truncated mutants of the N and P proteins were accomplished using a Q5 site-directed mutagenesis kit (NEB). The NiV N or P protein and truncation constructs were amplified by PCR, using the primers listed in Table 1.

### 2.2. Cell Culture

All cells were cultured at 37 °C in a humidified atmosphere with 5% CO_2_. A549 (ATCC CCL-185), U251 (Cell Bank of the Chinese Academy of Sciences TCHu 58), Vero (ATCC CRL-1587), Huh.7 (JCRB JCRB0403), HeLa (ATCC CCL-2) and HEp-2 (ATCC CCL-23) were cultured in Dulbecco’s modified Eagle’s medium (Gibco, C11995500BT) supplemented with 10% fetal bovine serum (FBS) (Invitrogen) and 1% penicillin–streptomycin (Life Technologies, Carlsbad, CA, USA).

### 2.3. Plasmid Transfection and Measurement of Reporter Gene Expression

A549 cells were seeded in a 96-well plate (1.8 × 10^4^ cells per well), cultured overnight, and then infected with the recombinant vaccinia virus (vTF7-3, ATCC VR-2153) at a multiplicity of infection (MOI) of 1 PFU/cell, and virus was allowed to adsorb at room temperature for 1 h. The vTF7-3 infected cells were then transfected with minigenome plasmid (88 ng) together with helper plasmids N (44 ng), P (22 ng), and L (44 ng). The transfection was performed using Lipofectamine 2000 (Invitrogen, Waltham, MA, USA) according to the manufacturer’s protocol. The transfected cells were incubated at 37 °C and tested at 6, 24, 48, and 72 h with an Olympus microscope (Olympus, Nagano Prefecture, Japan) or luciferase assay.

### 2.4. Luciferase Assay

The One-Lite^TM^ Luciferase Assay kit (Vazyme, Nanjing, China) was used to measure the luciferase activity. The cell culture plate was removed from the incubator and equilibrated at room temperature for 30 min. An equal volume of One-Lite™ detection reagent, which had been pre-equilibrated to room temperature, was subsequently added to the cell culture. After incubation at room temperature for 5 min to ensure complete cell lysis, the cell lysate was transferred to a 96-well white microplate, and Fluc activity was measured using enzyme-labeled instrument (PerkinElmer Ensight, Waltham, MA, USA).

### 2.5. RNA Isolation, RT-PCR, and RNA Quantification

Total cellular RNA was isolated using TRIzol reagent (Invitrogen, Carlsbad, CA, USA). NiV N/P/L RNA levels were measured by RT-qPCR and expressed as the RNA level relative to the GAPDH level. RT-PCR was performed using a HiScript II One Step qRT-PCR SYBR Green Kit following the protocol of the manufacturer (Vazyme, Nanjing, China). The RT-PCR primers for NiV N/P/L RNA were as indicated in Table 2. To detect the different forms of NiV RNAs, RT-qPCR was performed using tagged-strand-specific primers. Briefly, first-strand cDNAs were generated using tagged-strand-specific primers with a HiScript III first-strand cDNA Synthesis Kit (Vazyme), followed by strand-specific RT-qPCR detection. Quantitative PCR was carried out utilizing Fast start Universal SYBR Green Master (ROX) (Roche, Basel, Switzerland). The RT-qPCR primers for specific RNA were as indicated in Table 3.

### 2.6. Gene Silencing with siRNAs

The si90 targeting the 5′ UTR of the N gene and the si128 targeting the 3′ UTR of the L gene were designed using siRNA (Genepharma, Suzhou, China). Their target sequences were as follows: for si90 (5′-GGAUCCUCAAGAAAUAUAUTTAUAUAUUUCUUGAGGAUCCTT-3′); si128 (5′-CCAAGACCAACUGAUAACUTTAGUUAUCAGUUGGUCUUGGTT-3′). The siRNA targeting irrelevant sequence was used as a negative control (siNC 5′-UUCUCCGAACGUGUCACGU-3′). The NiV minigenome replicon and siRNA were co-transfected into a 96-well plate (1.8 × 10^4^ cells per well). The medium was changed to fresh DMEM supplemented with 2% FBS at 6 h post transfection (hpt). The antiviral effect of siRNA was measured on 24 hpt by qRT-PCR.

### 2.7. Interferon Treatment

IFN-α 2a (UA BIOSCIENCE, Nanjing China), IFN-γ (UA BIOSCIENCE, China), and IFN-λ 3 (R&D Systems, Minneapolis, MN, USA) were serially diluted to different concentrations with DMEM supplemented with 2% FBS to the indicated concentrations and added to A549 cells seeded in 96-well plates and cultured for 24 h before transfection. The cell lysates were harvested at 48 hpt for observation, measuring Fluc activity and RNA levels.

### 2.8. Drug Treatment

For the drug inhibition assays, A549 cells were seeded in a 96-well plate and cultured overnight before treatment. Azvudine, remdesivir, and molnupiravir (MedChem-Express, Monmouth Junction, NJ, USA) were dissolved in DMSO (Sigma, Livonia, MI, USA) and diluted to different concentrations with DMEM supplemented with 2% FBS immediately before use. The NiV minigenome replicon was transfected into vTF7-3 infected A549 cells. The fresh culture medium was replaced with the medium containing drugs or DMSO at 6 hpt. Cells were harvested and lysed at 48 hpt. DMSO (0.1%) was used as a control.

### 2.9. Confocal Fluorescence Microscope

Cells cultured on a glass-bottom cell culture dish (NEST, Wuxi, China) in DMEM supplemented with 10% FBS were transfected with pCAGGS-NiV-N-mCherry plasmid and pCAGGS-NiV-P-EGFP plasmid (or truncated mutant plasmids). Then, 48 h after transfection, cells were fixed with 4% paraformaldehyde (PFA) for 30 min. For labeling nuclei, we incubated them for 15 min with the addition of Hoechst 33,342 (Invitrogen). Cells were observed with a Nikon A1 confocal microscope (Nikon, Tokyo, Japan).

### 2.10. Fluorescence Recovery After Photobleaching

Fluorescence recovery after photobleaching (FRAP) analysis of condensates was performed using a Nikon A1 confocal microscope with a 100×/1.42 oil objective. The intensity of the fluorescent is controlled in the detection range through changing the laser power, digital gain, and offset. For green and red fluorescent channels, bleaching was conducted by 488 or 561 lines, correspondingly, and the laser power and iteration of bleaching were optimized to achieve an efficient bleaching effect. Fluorescence recovery was monitored at 2 or 4 s intervals for 5 min. In the focal-bleach experiment, roughly half (partial bleach) or all (full bleach) of a condensate was photobleached to determine the molecular mobility with diffuse pools or inside a condensate.

A549, U251, and Vero cells for FRAP experiments were cultured on a glass-bottom cell culture dish (NEST) in DMEM supplemented with 10% FBS and 1% penicillin–streptomycin.

The FRAP data were quantified using a Nikon A1 built-in profile model. The time series of the fluorescence intensity of condensates were calculated. The intensity of the condensate during the whole experiment was normalized to the one before bleaching, and the intensity of the granule just after bleaching was normalized to zero. At least 3 condensates per condition were analyzed to calculate the mean and standard deviation. The averaged relative intensity and standard error were plotted to calculate dynamics.

### 2.11. Live Cell Imaging

N-mCherry and P-EGFPs were co-expressed in A549, U251, and Vero cells and imaged directly with the Nikon A1. Single cells that expressed N-mCherry and P-EGFPs were imaged every 2.5 s. Two IBs that fused into one IB were marked and amplified.

### 2.12. Sensitivity of IBs to 1,6-Hexanediol Treatment

A549, U251, and Vero cells were inoculated into glass-bottomed cell culture dishes and co-transfected with N-mCherry and P-EGFP plasmid. Then, 48 h after transfection, 1.5% 1,6-hexanediol (1,6-HD) (*w*/*v*) was added to the cells. Image acquisition was performed using a Nikon A1 confocal microscope. Cells were stored in a climate-controlled chamber (37 °C, 5% CO_2_). Imaging was performed every 30 s for 2 min after treatment.

### 2.13. Statistical Analysis

GraphPad Prism 8.4 software (GraphPad Software Inc, San Diego, CA, USA) was used for statistical analysis. The data are expressed as the mean ± SD. All experiments were performed in triplicate, and a minimum of 3 independent experiments were evaluated. The differences between the experimental and control groups were determined by Student’s *t*-test for comparing two groups of data. *p* < 0.05 was considered to indicate statistical significance.

## 3. Results

### 3.1. Generation of the NiV Minigenome Replicon

**Figure 1:**
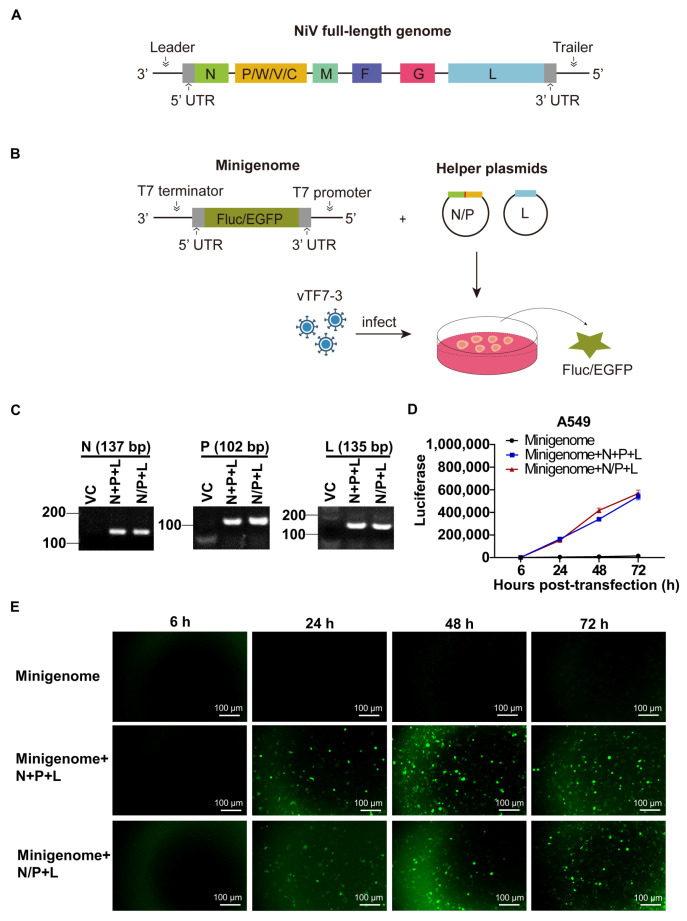
Generation of the NiV minigenome replicon. (**A**) Schematic representation of NiV full-length genome. (**B**) Schematic representation of NiV minigenome transient-transfection replicon. (**C**) RT-PCR analysis of N+P+L or N/P+L gene RNA expression. (**D**,**E**) A549 cells were infected with vTF7-3 for 1 h, then the NiV minigenome replicon was transfected into the cells, and cells were harvested at the indicated time for analysis. (**D**) Detection of luminescence in the NiV Fluc minigenome replicon. (**E**) The expression of EGFP in A549 cells transfected with the NiV EGFP minigenome replicon was monitored. The error bar represents the standard deviations of the results of three independent experiments.

To develop an NiV research tool that was both operable and suitable for drug screening in the BSL-2 laboratory, the NiV minigenome replicon was constructed. As shown in Figure 1A,B, the NiV minigenome replicon comprises three plasmids: (1) a T7 promoter-driven construct containing the N 5′ UTR (60 bp), a reporter gene (FLuc or EGFP), the L 3′ UTR (70 bp), and a T7 terminator; (2) N and P plasmids or a bicistronic N/P plasmid; and (3) an L-expressing plasmid. The antisense-orientated minigenome cDNA prevents host RNA polymerase-driven transcription, while the replacement of the native open reading frame with reporter genes preserves essential cis-acting replication elements (Figure 1A,B). We first analyzed the expression levels of the N, P, tandem N/P and L plasmids (Figure 1C). Subsequently, the NiV minigenome plasmid was transfected into vTF7-3-infected A549 cells alone; with plasmids encoding N, P, and L, respectively; or with plasmids encoding N/P and L, respectively, to evaluate its replication and transcription ability. No significant differences were observed in the replication and transcription efficiency of the minigenome between the three-plasmid and four-plasmid systems, as measured by Fluc activity at 6, 24, 48, and 72 hpt (Figure 1D). Cells transfected solely with the minigenome plasmid, which served as a negative control, exhibited no detectable activity. The results obtained from the EGFP minigenome replicon were comparable to those of the Fluc minigenome replicon. During the experimental period, the EGFP expression exhibited a time-dependent increase, as demonstrated in Figure 1E. No significant difference was observed between the three-plasmid and four-plasmid systems. As a negative control, cells transfected with only the minigenome plasmid exhibited no detectable EGFP fluorescence signal. These results suggest that the NiV minigenome can undergo transcription and replication with the assistance viral proteins N, P, and L. Although the three-plasmid system does not significantly enhance efficiency compared to the four-plasmid system, it simplifies the transfection procedure.

### 3.2. LLPS Drives the Formation of NiV IB-like Structures

**Figure 2:**
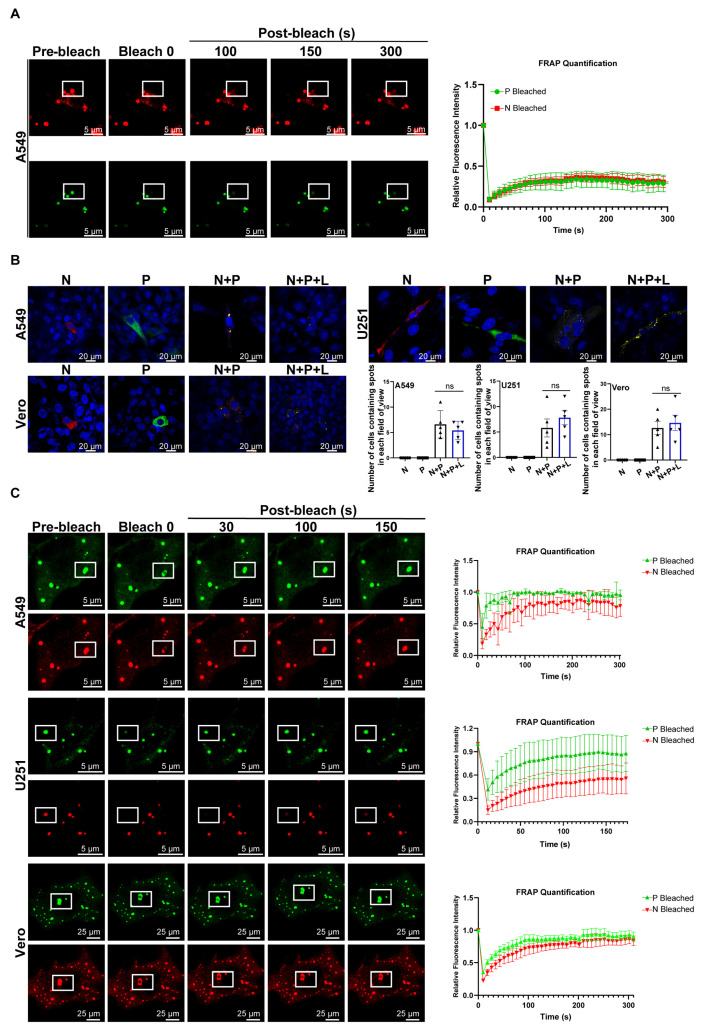

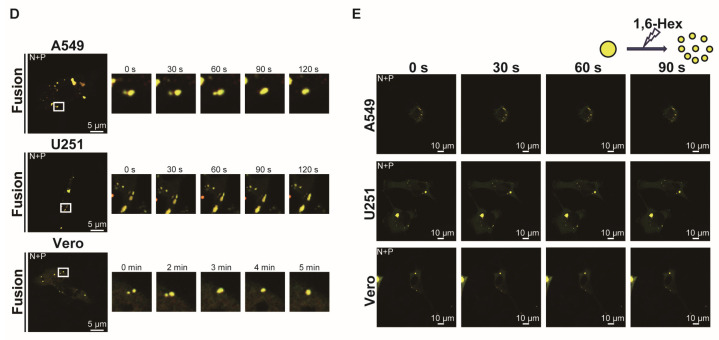
LLPS drives the formation of NiV IB-like structures. (**A**) NiV Fluc minigenome plasmid and helper plasmids encoding NiV N-mCherry, P-EGFP, and L proteins were transfected into vTF7-3-infected A549 cells. FRAP images of IBs to indicate the internal diffusion and diffusion across boundary properties. Three NiV IBs were bleached with strong 488 nm and 568 nm laser pulse. Images were captured every 4 s. The right histogram shows relative fluorescence intensities of bleached IBs. These live cells proceeded to undergo time series imaging 48 hpt. The white frame demarcates the region exhibiting IB fluorescence quenching. (**B**) NiV N protein and P protein are the minimal components for the formation of IBs. The N-mCherry plasmid and P-EGFP plasmid are transfected individually, the N-mCherry and P-EGFP plasmids are co-transfected, and the N-mCherry, P-EGFP, and L plasmids are co-transfected in A549, U251, and Vero cells, respectively. The distribution of viral proteins was observed by fluorescence microscopy. The statistical results quantifying IB formation across different transfection systems are shown in the lower right corner, with each experimental group containing five analyzed microscopic fields. Statistical significance was analyzed by Student’s *t*-test (ns, not significant). (**C**) N-mCherry and P-EGFP plasmids were co-expressed in A549, U251, and Vero cells, respectively. This experiment was performed as described above in (**A**). (**D**) Representative images of two bodies fusing into one. Images were captured every 2 s. (**E**) The 1,6-HD compound was used to treat EGFP-tagged P and mCherry-tagged N-expressing live cells. Images were captured every 2 s.

Next, we examined the formation of IB-like structures in A549 cells following transfection with the NiV minigenome replicon, in which the N/P plasmid was replaced by the N-mCherry and P-EGFP plasmids. The IBs’ point-like aggregation structure was successfully detected at 24 hpt of the NiV minigenome replicon; FRAP experiments showed the limited molecular exchange capacity between IBs and the cytoplasm (Figure 2A). The minimal elements of NiV IB formation were then examined. As shown in Figure 2B, NiV-N-mCherry or NiV-P-EGFP plasmid transfection alone results in their diffuse distribution in A549, U251, or Vero cells. While the co-transfection of N-mCherry and P-EGFP plasmids induced the formation of IBs that strongly co-localized with N-mCherry and P-EGFP, the co-expression of N-mCherry, P-EGFP, and L did not increase the amount of IBs (Figure 2B). These results indicate that the NiV N and P proteins constitute the minimal elements required for the formation of IBs.

We then explored the physical characteristics of IBs. FRAP assays were performed with the co-transfection of N-mCherry and P-EGFP plasmids in A549, U251, and Vero cells. The results showed that the fluorescence within the IBs rapidly recovered shortly after photobleaching. This observation suggests that N and P proteins in the IBs undergo swift molecular exchange with proteins in the cytoplasmic milieu (Figure 2C). To further elucidate the dynamic behavior of IBs, live-cell imaging was performed 48 hpt in these cells. The results demonstrated that adjacent IBs fused into a single structure, revealing their liquid-like properties (Figure 2D). This evidence suggests that NiV IBs are formed through LLPS, a process driven by weak, transient interactions between multivalent or intrinsically disordered protein domains. 1,6-HD is known to disrupt LLPS-dependent condensates by interfering with hydrophobic interactions. However, IBs induced by NiV minigenome replicon were resistant to 1,6-HD, suggesting that the predominant interactions underlying the formation of NiV liquid-like IBs are not of a hydrophobic nature (Figure 2E). Collectively, these results demonstrate that the NiV minigenome replicon is capable of forming IBs and that NiV IBs are driven by LLPS.

### 3.3. Identification of the Domains of P and N Required for the Morphogenesis of IBs

**Figure 3:**
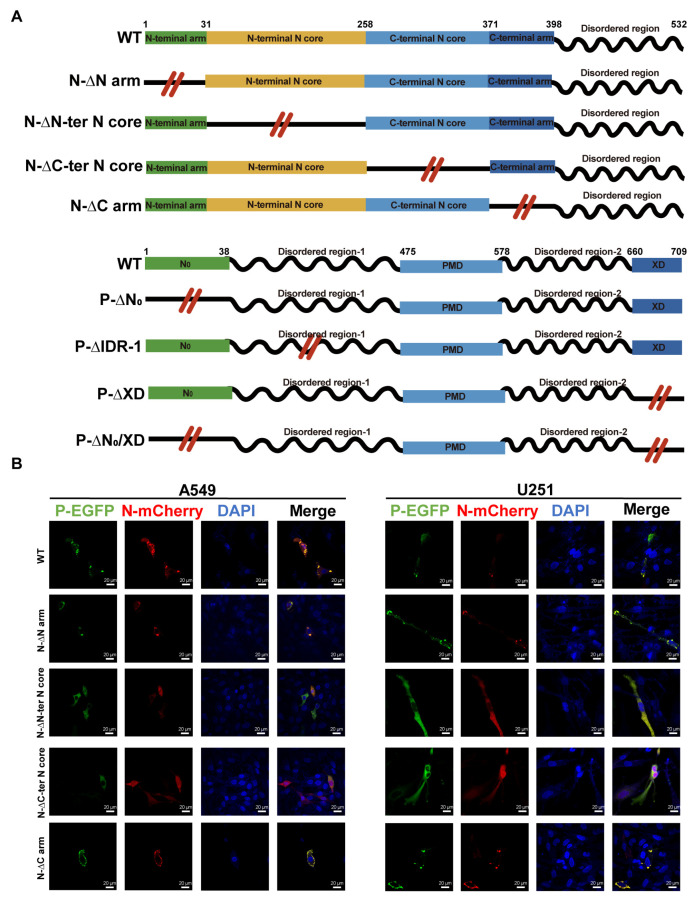

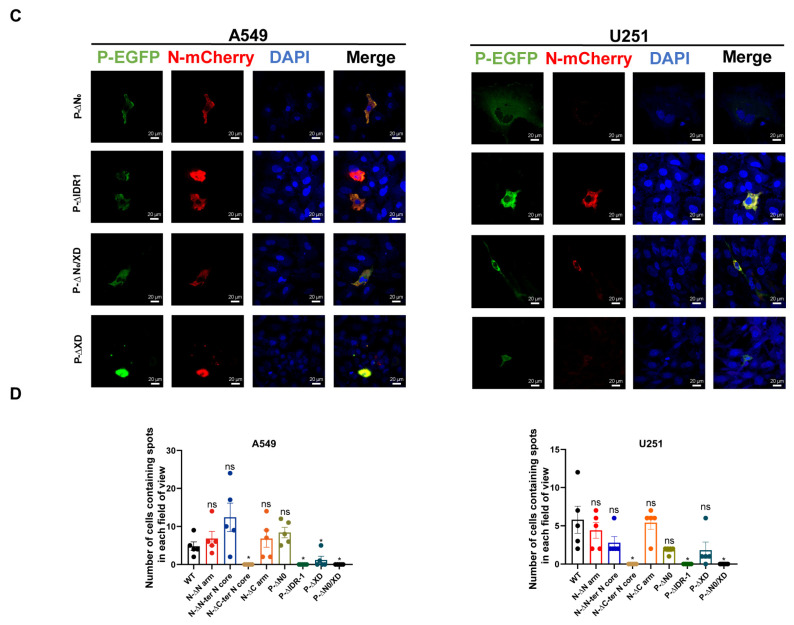
Identification of the domains of P and N required for the morphogenesis of IBs. (**A**) Schematic illustration of truncated P and N protein. NiV N and P protein is composed of modular domains (boxes) connected by disordered linkers (lines). N-terminal region (1–38) of P protein facilitates chaperoning of N_0_. Residues 475–578 of the P protein constitute the P multimerization domain (PMD). Residues 660–709 of the P protein constitute the C-terminal X domain (XD), which binds the nucleocapsid. (**B**,**C**) N and P proteins or their truncated forms were co-expressed in A549 and U251 cells. The cells were then fixed 48 hpt, and the distribution of viral proteins was observed by fluorescence microscopy. (**B**) Cellular localization of N truncated and P in cells. (**C**) Cellular localization of N and P truncated in cells. (**D**) Statistical analysis of IB-positive cells generated by P, N, or their truncated proteins. The error bars represent standard deviations of results of five independent fields of view. Statistical significance was analyzed by Student’s *t*-test (* *p* < 0.05; ns, not significant).

Based on previous structural analyses [25,26], to define the critical domain of N and P for NiV IBs, the domain-truncated N and P were constructed as shown in Figure 3A. Plasmid expression analysis confirmed that the various constructs exhibited no differences in expression levels (Figure S1). Analysis by fluorescence confocal microscopy revealed the subdomain requirements for IB assembly in A549 or U251 cells. The results showed that the deletion of the C-terminal N core domain of the N protein (N-ΔC-ter N core) or the first intrinsically disordered region (P-ΔIDR-1) of the P protein abolished the formation of IBs (Figure 3B–D). The deletion of the C-terminal X domain (P-ΔXD) of the P protein in A549 cells significantly impaired the formation of IBs. Only the deletion of both the N_0_ and XD of the P protein prevented the formation of IBs in A549 and U251 cells (Figure 3C,D). The observed differences in A549 and U251 cells highlight the complexity and variability of host factors involved in P-XD domain-mediated IB formation, although it is not yet clear why P-XD domain-mediated IB formation varies between cells. Overall, these results demonstrate that the N-C-terminal N core, P-N_0_, P-XD, and P-IDR-1 domains are essential components for NiV IB assembly. Additionally, we observed the formation of semi-solid, non-spherical condensates in the P-ΔXD and P-ΔIDR1 of A549 cells, as well as in the P-ΔIDR1 of U251 cells.

### 3.4. The NiV Minigenome Replicon Is Sensitive to RNA Interference and Interferons

**Figure 4:**
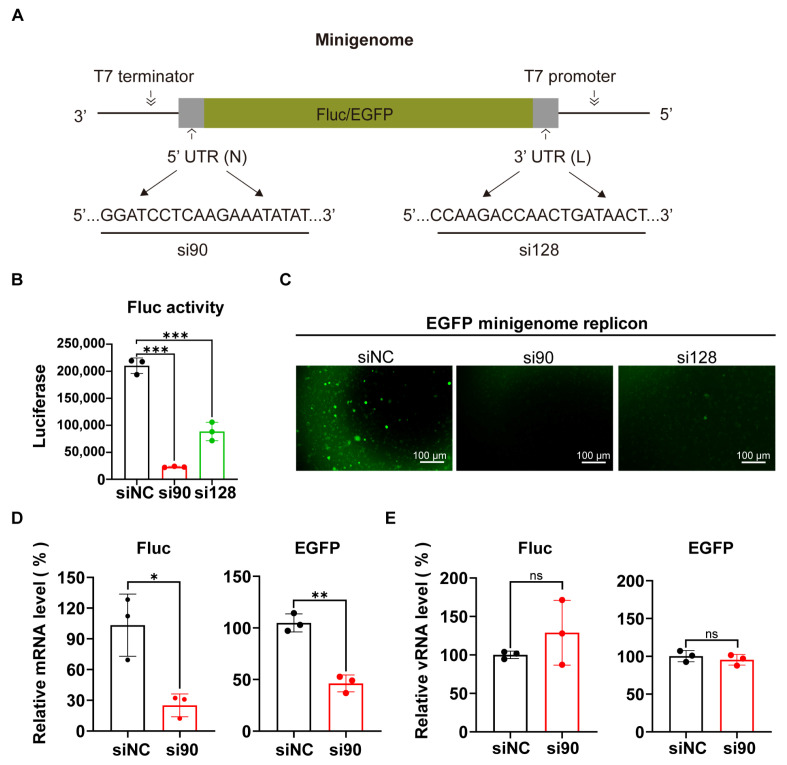
The NiV minigenome replicon is sensitive to RNA interference. (**A**) Schematic representation of the two NiV-specific siRNA targeting sites. (**B**–**E**) A549 cells were infected with vTF7-3 for 1 h, then the NiV minigenome replicon and siRNA were co-transfected into the cells, and cells were harvested at 24 hpt for analysis. The levels of Fluc activity (**B**), EGFP expression (**C**), mRNA (**D**), and vRNA (**E**) were measured. All the data are expressed as percentages of siNC treatment. The error bars represent standard deviations of results of three independent experiments. Statistical significance was analyzed by Student’s *t*-test (* *p* < 0.05, ** *p* < 0.01, *** *p* < 0.001; ns, not significant).

To test whether the NiV minigenome replicon is sensitive to RNA interference, we first evaluated two interference RNAs, one (si90) targeting the 5′ UTR of the NiV N gene and the other (si128) targeting the 3′ UTR of the NiV L gene in the NiV minigenome (Figure 4A). The Fluc activity or EGFP expression was measured after the Fluc-minigenome or EGFP-minigenome was interfered with using si90, si128, or siRNA negative control (siNC) in vTF7-3-infected A549 cells, respectively. The results showed that both si90 and si128 significantly reduced Fluc activity (Figure 4B), and similar results were also detected in the EGFP-minigenome replicon (Figure 4C). Consistently, the knockdown of the NiV minigenome effectively decreased the mRNA level of the reporter gene (Figure 4D). Interestingly, si90 could not reduce vRNA levels (Figure 4E), mirroring observations in Zhong’s Ebola minigenome replicon [12]. This supports the hypothesis that vRNA is shielded by viral RNPs.

**Figure 5:**
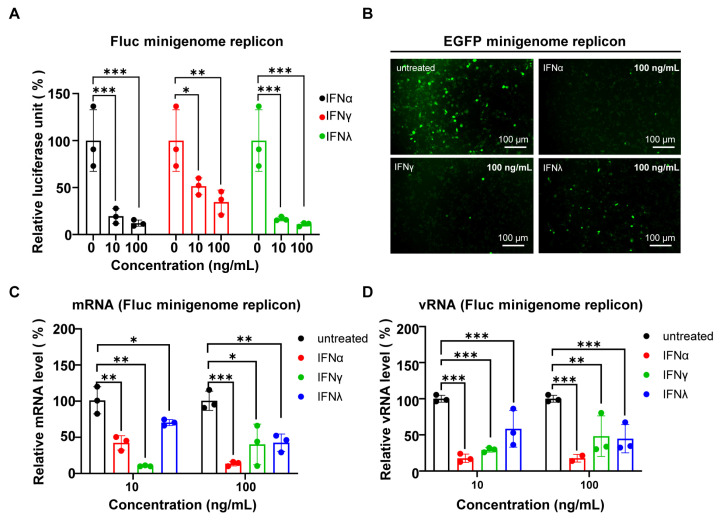
NiV replication in the minigenome replicon was sensitive to interferon treatment. (**A**–**D**) NiV minigenome replicons were treated with different concentrations of IFN-α, IFN-γ, and IFN-λ. Inhibitory effects were quantified by Fluc activity, fluorescence signals, and RT-qPCR analysis of NiV mRNA and vRNA and expressed as percentages of no treatment as a control. The Fluc activity (**A**) and the expression of EGFP (**B**) were detected at 48 h after IFN treatment. The mRNA (**C**) and vRNA (**D**) levels of the NiV minigenome replicon were quantified by RT-qPCR and expressed as percentages of control results. The error bars represent standard deviations of results of three independent experiments. (* *p* < 0.05, ** *p* < 0.01, *** *p* < 0.001).

Next, we analyzed whether the NiV minigenome replicon was sensitive to different interferons (IFNs). The NiV minigenome replicon-transfected A549 cells were treated with IFN-α 2a (type I), IFN-γ (type II), and IFN-λ 3 (type III) at indicated doses for 48 h (Figure 5A–D). As shown in Figure 5A, both the transcription and replication of the NiV minigenome replicon were inhibited in a dose-dependent manner, as evidenced by the measurement of the Fluc activity of the NiV minigenome. Similarly, the expression of EGFP was markedly suppressed by treatment with 100 ng/mL IFNs in the EGFP minigenome replicon system (Figure 5B). Furthermore, following IFN treatment, a substantial decrease was observed in both the NiV minigenome replicon mRNA and vRNA levels (Figure 5C,D). Collectively, these results demonstrate that the NiV minigenome replicon was sensitive to different IFNs.

### 3.5. Evaluation of Antiviral Drugs Using NiV Minigenome Replicon

**Figure 6:**
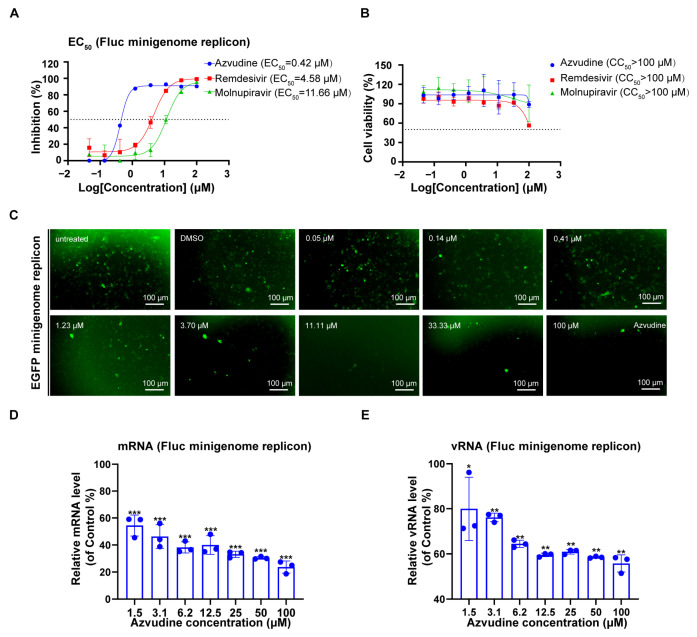
Evaluation of antiviral drugs using NiV minigenome replicon. (**A**,**B**) Antiviral activity of azvudine, remdesivir, and molnupiravir against NiV Fluc minigenome replicon in luciferase assay. The vTF7-3 infected A549 cells were treated with azvudine, remdesivir, and molnupiravir at the indicated concentration for 48 h. Fluc activity and cell viability of each group were used to calculate the EC_50_ (**A**) and CC_50_ (**B**) with GraphPad software, respectively. (**C**) The EGFP expression was tested at different concentrations after azvudine treatment in the NiV EGFP minigenome replicon. The mRNA (**D**) and vRNA (**E**) levels were quantified by RT-qPCR and expressed as percentages of control results after azvudine treatment in the NiV Fluc minigenome replicon. The error bars represent the standard deviations of results of three independent experiments. Statistical significance was analyzed by Student’s *t*-test (* *p* < 0.05, ** *p* < 0.01, *** *p* < 0.001).

Furthermore, we used the NiV minigenome replicon to evaluate the anti-NiV transcription and replication activity of three broad-spectrum RdRp-targeting nucleic acid analogs. The vTF7-3 infected A549 cells were pre-incubated with these compounds for 2 h and then transfected with the NiV Fluc minigenome replicon. At 48 hpt, the cytotoxicity and Fluc activity were measured. We found that the treatment of azvudine, remdesivir, and molnupiravir could decrease the Fluc activity in a dose-dependent manner, with EC_50_ values of 0.42, 4.58, and 11.66 μM without affecting cell viability in A549cells, respectively (Figure 6A,B). Furthermore, azvudine decreased the GFP level when used in the NiV EGFP minigenome replicon (Figure 6C). Notably, azvudine dose-dependently decreased both NiV mRNA and vRNA levels in the NiV Fluc minigenome replicon, confirming its potent inhibitory effect on NiV transcription and replication (Figure 6D,E). Collectively, these results also indicated the feasibility of using the NiV minigenome replicon to screen anti-NiV transcription and replication drug inhibitors.

We next analyzed whether azvudine could inhibit the NiV Fluc minigenome replicon by affecting the formation of IBs of NiV N, P, and L proteins. The results showed that azvudine treatment did not interfere with the formation of NiV IBs (Figure S2). This result suggested that azvudine might inhibit NiV minigenome replicon by acting as a nucleoside analog to affect the process of RNA synthesis but not by disrupting IB formation by interfering with the interactions among N, P, and L proteins.

## 4. Discussion

The NiV, a highly pathogenic zoonotic paramyxovirus, poses significant public health risks due to its high mortality rate (40–70%) [1]. There is currently no clinically approved drug or vaccine for NiV infection. Traditional antiviral drug discovery approaches, which rely on live virus infection, encounter significant challenges, including biosafety restrictions, ethical concerns, and technical complexity [27]. Here, we developed two NiV minigenome replicons based on Fluc and EGFP, respectively, which allowed for the study of replication mechanisms and drug screening under BSL-2 conditions. Notably, we initially observed that the minigenome replicon can mimic the cytoplasmic IBs formation observed in live NiV infections, where IB assembly is driven by LLPS. Importantly, the minigenome replicon is sensitive to type I/II/III IFNs and antivirals (remdesivir, azvudine, molnupiravir).

The minigenome replicon can reduce reliance on ethically challenging animal models and resource-intensive BSL-4 workflows, democratizing global research efforts to combat this high-risk pathogen. By incorporating reporter genes, it allows the high-throughput quantification of replication/transcription inhibition, accelerating the identification of RdRp-targeting compounds [28]. Although NiV minigenome replicon studies have been reported, there also have some differences. Compared with other transient transfection systems [11,14,29], we reduced the number of transfected plasmids and simplified the transfection system (Figure 1). In contrast with Ke’s NiV stable transfection system [11], our transient transfection approach eliminates dependency on engineered stable cell lines, thereby enabling functional studies in NiV tropic cell models that more faithfully recapitulate the virus’s native transcription and replication machinery.

In mammalian cells, UTRs function as regulatory hubs that recruit multiple factors involved in post-transcriptional gene regulation [30]. Kimihiro Hino et al. demonstrated that the 3′ UTR of the NiV N gene orchestrates post-transcriptional regulation [31]. Shotaro Uchida et al. revealed that the 5′ UTR of the NiV M gene enhanced the translation of a reporter gene [32]. Our results showed that siRNA targeting the 5′ UTR of NiV N gene or 3′ UTR of the NiV L gene displayed a decent ability to decrease the minigenome expression and mRNA level (Figure 4). Nevertheless, these siRNAs have no effect on vRNA. Prior research posited that this phenomenon may be attributed to the protective function of the vRNP complex [12,33,34]. The therapeutic relevance of UTR-targeting strategies is further supported by IFN studies. Type I IFNs are pivotal in conferring murine resistance to lethal NiV infection [35], while type III IFNs (IFN-λ) specifically inhibit NiV replication in respiratory epithelia [36]. Our data extend these observations, showing that all type I-III IFNs suppress NiV minigenome activity, corroborating their broad antiviral potency against viral RNA synthesis (Figure 5). Furthermore, we identified azvudine as a potent inhibitor of NiV replication using the minigenome replicon and found that remdesivir, and molnupiravir also exerted an inhibitory effect on the replication of this virus (Figure 6). This is consistent with live virus research findings, demonstrating that nucleoside analog drugs such as remdesivir effectively inhibit the NiV genome replication [37]. Further investigation into the mechanisms of these drugs in combating NiV infection is essential.

Viral IBs, membrane-less organelles formed via LLPS, serve as critical replication hubs by concentrating viral machinery and enabling efficient RNA synthesis [38]. Since Hyman’s landmark discovery in liquid–liquid phase separation [39], IBs have been recognized as dynamic sites where de novo viral RNA production occurs within RNP-rich condensates [40]. We observed the formation of IB-like structures exhibiting a point-like aggregation pattern in A549 cells following transfection with the NiV minigenome replicon (Figure 2A). Analysis conducted in Vero (NiV propagation), A549, and U251 (NiV-permissive) cells demonstrated that both the N and P proteins are indispensable in the assembly of IBs. The co-expression of N and P proteins induces IB formation in multiple cell lines, including A549, U251, Vero, Huh7, HeLa, and HEp-2. While Huh7, HeLa, and HEp-2 cells showed higher transfection efficiency, IB-positive cell counts remained comparable (Figure S3). LLPS arises from multivalent interactions between proteins and/or RNAs, driving the formation of membrane-less condensates critical to cellular organization. Central to this process are IDRs in proteins, which enable weak, transient interactions that promote phase separation [41,42]. Strikingly, live-cell imaging and FRAP revealed that NiV IBs exhibit liquid-like properties (Figure 2C,D), providing the first direct evidence of LLPS-driven IB biogenesis in NiV. However, not all LLPS depends on IDRs—structured oligomerization domains, as seen in SARS-CoV-2 ORF8, can also mediate phase-separated droplets [43]. 1,6-HD, a chemical disruptor of hydrophobic interactions, dissolves LLPS-driven phase-separated droplets, as demonstrated in HIV latency (e.g., CBX4-EZH2 nuclear body) [44] and MeV IBs [45]. Our results demonstrate that NiV IBs exhibit resistance to 1,6-HD treatment, suggesting that the primary interactions mediating the formation of NiV N-P protein-induced IBs are not predominantly hydrophobic (Figure 2E). This resilience highlights NiV’s unique adaptation: while canonical LLPS relies on IDR-driven weak interactions, NiV IBs may employ structured oligomerization or stronger binding interfaces to maintain replication hubs.

In summary, we generated a novel NiV minigenome replicon and modeled the phenomenon of IB generation based on the NiV minigenome replicon, providing a useful tool for studying NiV replication and screening anti-NiV compounds under BSL-2 conditions.

## Figures and Tables

**Table 1:** Primers for NiV plasmid construction.

Primer	Sequence (5′-3′)
N-mCherry-F	GGCCGACGTGGGAAGCGGAAGCGGAAGCATGGTGAGCAAGGGCGAGG
N-mCherry-R	TTACTTATCGTCGTCATCCTTGTAATCCTTGTACAGCTCGTCCATGCC
P-EGFP-F	CGGTAACATCGGAAGCGGAAGCGGAAGCATGGTGAGCAAGGGCGAG
P-EGFP-R	GGCATGGACGAGCTGTACAAGGATTACAAGGATGACGACGATAAGTAA
N-N-arm-del-F	ACCACCAAGATCCGGATCT
N-N-arm-del-R	AATTCTTTGCCAAAATGATGAGACAGCAC
N-N-Core-del-F	GTGGAGGAGACCGGCAT
N-N-Core-del-R	CGCCACCGCCACCCTG
N-C-Core-del-F	GGGGGGATTGACCAGAACATGG
N-C-Core-del-R	GGGGGGATTGACCAGAACATGG
N-C-arm-del-F	ACCAGCGCCGGGAGACAGG
N-C-arm-del-R	GTGATGCCTGGCGCTCTTCT
P-N_0_-del-F	AAGGACCAGACCAAGGCTTGG
P-N_0_-del-R	TGCTGTCTCATCATTTTGGCAAAGAATT
P-IDR1-del-F	TGCTGTCTCATCATTTTGGCAAAGAATT
P-IDR1-del-R	ATCCATCCAGCAGCCAAGCATC
P-XD-del-F	GGAAGCGGAAGCGGAAGC
P-XD-del-R	GGAAGCGGAAGCGGAAGC

**Table 2:** Primers for detecting the expression of Nipah virus proteins.

Target	Primer	Sequence (5′-3′)
N	N-qPCR-F	GACATCTTCGAGGAGGCCGCCTCCT
	N-qPCR-R	TCCCACCTCAGCTCGGGGCTATTGGT
P	P-qPCR-F	GCTGGTGAACGACGGTCTGAAC
	P-qPCR-R	TTGATGCTTGGCTGCTGGATGG
L	L-qPCR-F	ACCACAAGTACCGCCGCATTG
	L-qPCR-R	GCATCATGGAGCCACTACCTTCAC

**Table 3:** The RT-qPCR primers for specific RNA.

Primer	Sequence (5′-3′)
vRNA-F	GGCCGTCATGGTGGCGAAT
vRNA-EGFP-tag	GGCCGTCATGGTGGCGAATACGTAAACGGCCACAAGTTC
vRNA-Fluc-tag	GGCCGTCATGGTGGCGAATGACAAGGATGGATGGCTACATTCTG
EGFP-R	AAGTCGTGCTGCTTCATGTG
vFluc-R	GGGTGTTGGAGCAAGATGGATTC
mRNA-tag	CCAGATCGTTCGAGTCGT
mRNA-R	CCAGATCGTTCGAGTCGT
mFluc-F	CCCACAACCTCGTTCTACCTAAG
mEGFP-F	TTCAGCACGACGAAGTACAC

## Data Availability

All research data associated with the paper can be obtained upon request from Q.Y.

## Supplementary Materials

The following supporting information can be downloaded at: https://www.mdpi.com/article/10.3390/v17050707/s1. Figure S1. Detection of expression levels of N protein, P protein, and their respective truncated variants. Figure S2. Effect of azvudine treatment on the formation of IBs by NiV minigenome replicon. Figure S3. In a variety of cell lines, NiV N and P proteins can form IBs.

## Abbreviations

The following abbreviations are used in this manuscript:

NiV	Nipah virus
RSV	Respiratory syncytial virus
EBOV	Ebola virus
MARV	Marburg virus
MeV	Measles virus
vTF7-3	Recombinant vaccinia virus expressing bacteriophage T7 polymerase
BSL-4	Biosafety level 4
BSL-2	Biosafety level 2
Fluc	Firefly luciferase
EGFP	Enhanced green fluorescent protein
IDR	Intrinsically disordered region
LLPS	Liquid–liquid phase separation
UTR	Untranslated region
CAT	Chloramphenicol acetyltransferase
CV-1	African green monkey kidney fibroblast cells
IBs	Inclusion bodies
vRNPs	Viral ribonucleoproteins
RNP	Ribonucleoprotein
RdRp	RNA-dependent RNA polymerase
P2A	Porcine teschovirus-1 2A peptide
RT-qPCR	Reverse transcription quantitative polymerase chain reaction
FRAP	Fluorescence recovery after photobleaching
1,6-HD	1,6-hexanediol
IFN	Interferon
RNAi	RNA interference
EC_50_	Half-maximal effective concentration
CC_50_	Half-maximal cytotoxic concentration
ANOVA	Analysis of variance
