# Supplementary material for: Construction of Minigenome Replicon of Nipah Virus and Investigation of Biological Activity
Source: Viruses. 2025 May 15;17(5):707. doi: 10.3390/v17050707 (PMC12116097; doi:10.3390/v17050707)
Supplement: Supplementary file 1 [file viruses-17-00707-s001.zip › viruses-3596327-supplementary.pdf]

Supplementary Materials for

Construction of Minigenome Replicon of Nipah Virus and Investigation of Biological Activity

Fan Wang [1,2,†], Ruyi Chen [1,3,†], Jiayi Zhong [1,†], Anqi Zhou [1,4], Ran Peng [1,5], Bao Xue [1,3], Yuan Zhou [1], Jielin Tang [1,4,*], Xinwen Chen [1,4,*] and Qi Yang [1,4,*]

Correspondence to: Jielin Tang (tang_jielin@gzlab.ac.cn), Xinwen Chen (chen_xinwen@gzlab.ac.cn), Qi Yang (yang_qi@gzlab.ac.cn)

This PDF file includes:

Supplementary Figures S1–S3

Figure S1

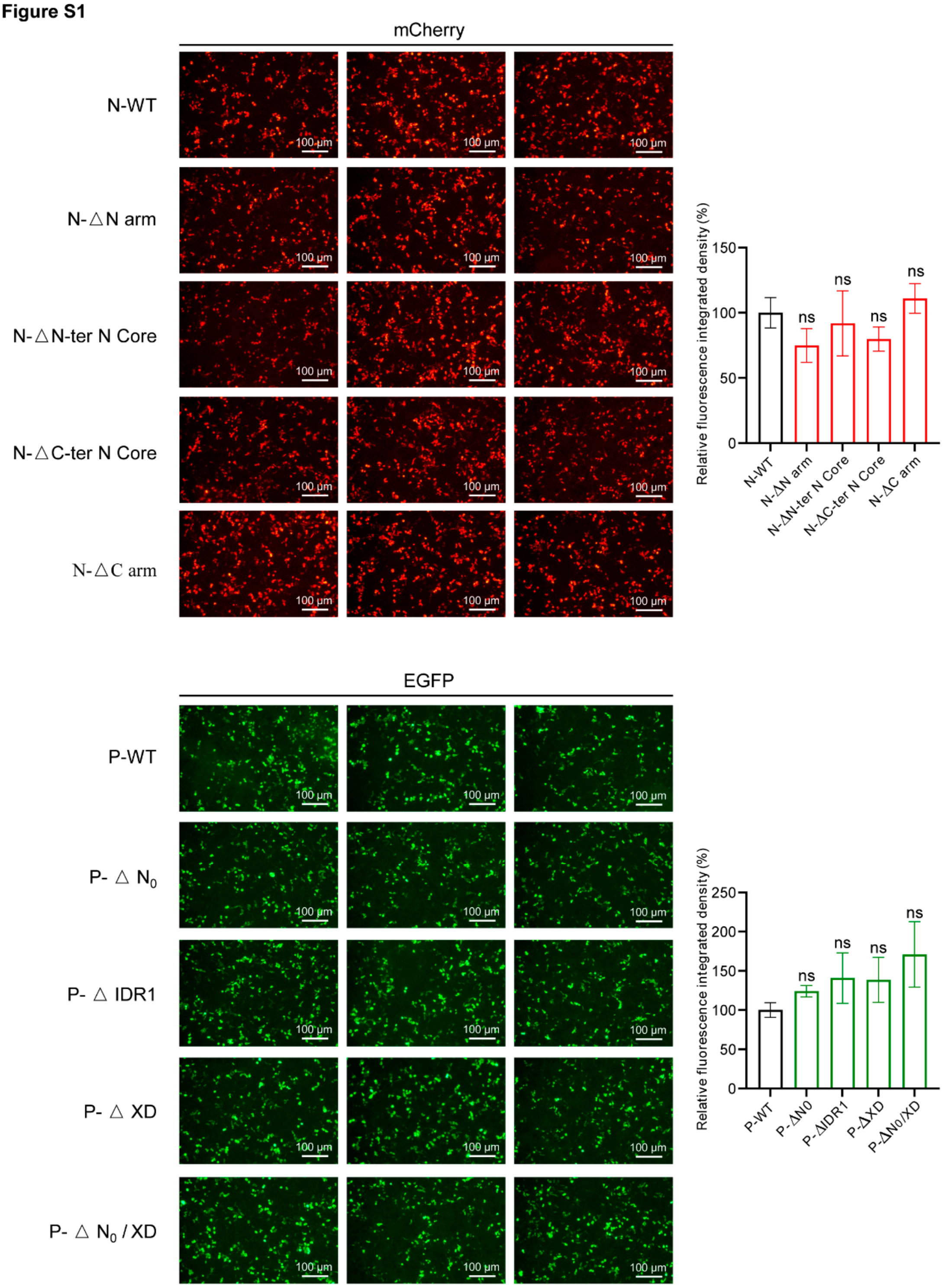

Figure S1. Detection of expression levels of N protein, P protein, and their respective truncated variants. We separately transfected HEK293T cells in 6-well plates with 1μg of plasmids encoding N protein, P protein, and their respective truncated variants. The results were observed under a fluorescence microscope 48 hours post-transfection. Fluorescence intensity per field was quantified using ImageJ software, and statistical significance was analyzed by Student's *t*-test (ns, not significant).

Figure S2

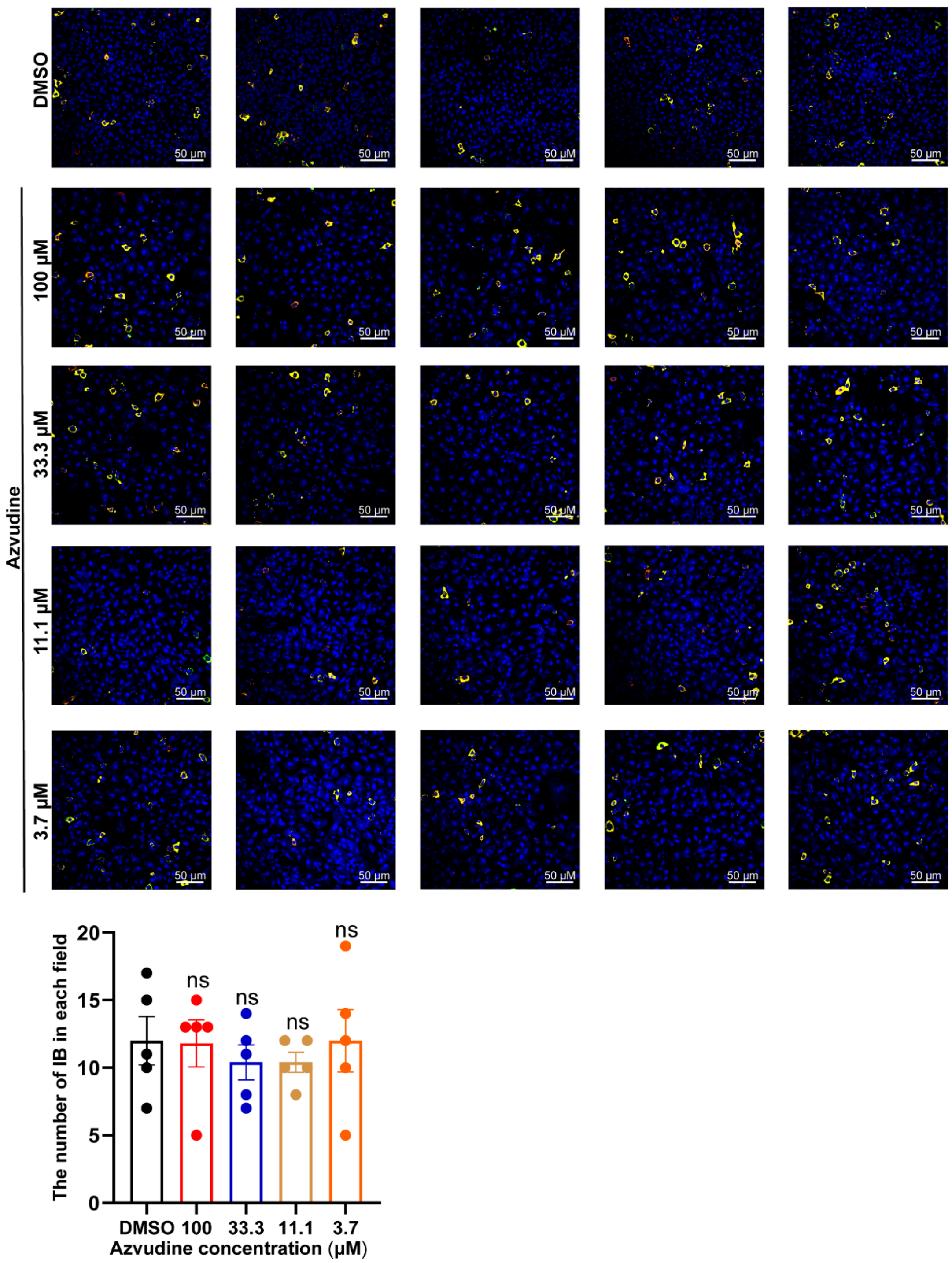

Figure S2. Effect of azvudine treatment on the formation of IBs by NiV minigenome replicon. NiV minigenome, along with helper plasmids individually expressing NiV N-mCherry, P-EGFP, and L protein, were co-transfected into A549 cells pre-infected with vTF7-3, a gradient dilution of azvudine was added 6 hours after transfection, and the number of IBs was observed under a fluorescence confocal microscope 48 hours later in groups of five fields of view. Count positive cells in five fields of view. The error bars represent standard deviations of results of five independent field of view. Statistical significance was analyzed by Student's *t*-test (ns, not significant).

Figure S3

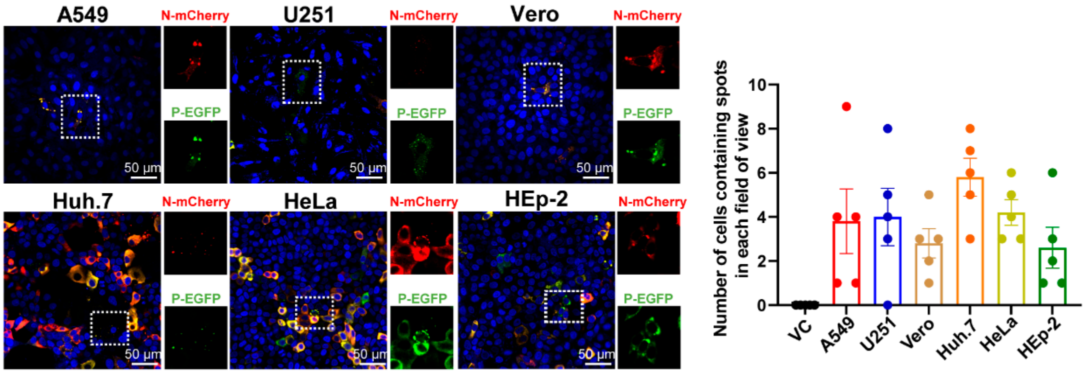

Figure S3. In a variety of cell lines, NiV N and P proteins can form IBs. Observation of cellular localization of N-mCherry and P-EGFP in cells. N-mCherry and P-EGFP plasmids were co-expressed in A549, U251, Vero, Huh.7, HeLa and HEp-2 cells respectively. The cells were then fixed 48 hpt, and the distribution of viral proteins was observed by fluorescence microscopy. Nuclei were stained with Hoechst 33342. The error bars represent standard deviations of results of five independent field of view.
